# Gene Ontology Function prediction in Mollicutes using Protein-Protein Association Networks

**DOI:** 10.1186/1752-0509-5-49

**Published:** 2011-04-12

**Authors:** Antonio Gómez, Juan Cedano, Isaac Amela, Antoni Planas, Jaume Piñol, Enrique Querol

**Affiliations:** 1 Institut de Biotecnologia i Biomedicina and Departament de Bioquímica i Biologia Molecular. Universitat Autònoma de Barcelona. 08193 Bellaterra, Barcelona. Spain; 2 Department of Systems Biology, Universitat de Vic, Vic, Spain; 3 Laboratory of Biochemistry, Institut Químic de Sarrià, Universitat Ramon Llull, 08017 Barcelona, Spain

## Abstract

**Background:**

Many complex systems can be represented and analysed as networks. The recent availability of large-scale datasets, has made it possible to elucidate some of the organisational principles and rules that govern their function, robustness and evolution. However, one of the main limitations in using protein-protein interactions for function prediction is the availability of interaction data, especially for Mollicutes. If we could harness predicted interactions, such as those from a Protein-Protein Association Networks (PPAN), combining several protein-protein network function-inference methods with semantic similarity calculations, the use of protein-protein interactions for functional inference in this species would become more potentially useful.

**Results:**

In this work we show that using PPAN data combined with other approximations, such as functional module detection, orthology exploitation methods and Gene Ontology (GO)-based information measures helps to predict protein function in *Mycoplasma genitalium*.

**Conclusions:**

To our knowledge, the proposed method is the first that combines functional module detection among species, exploiting an orthology procedure and using information theory-based GO semantic similarity in PPAN of the *Mycoplasma *species. The results of an evaluation show a higher recall than previously reported methods that focused on only one organism network.

## Background

Sequence similarity has proven to be useful for many years in attempting to annotate genomes [1,2]. A simple way to infer the possible function of a protein is to use an alignment procedure such as PSI-BLAST [3], to find possible homologues in sequence databases, such as UniProt [4]. However, sequence homology has its limitations. Only a fraction of newly discovered sequences have identifiable homologous genes in current databases, and its viability is limited to cases where substantial sequence similarity to annotated proteins can be found [5]. Moreover, the growing number of annotations extrapolated from sequence similarity is prone to errors [6-8], hence, new bioinformatics methods are developed to complement traditional sequence homology-based methods.

The development of high throughput technologies has resulted in large amounts of predicted Protein-Protein Interaction networks (PPI) for different genomes and, subsequently, methods using PPI data for functional inference [6,9-12] have been developed. It has been demonstrated that we may be able to use the semantics of gene annotations [13,14] and that we can obtain greater precision to predict new annotations using Gene Ontology (GO) information inside PPI [9,10,15]. Several semantic similarity measures using the GO database have been applied to gene products annotated with high ratios of prediction accuracy [13,15-19].

Recently, other methods using PPI to predict functions for individual genes or proteins have been developed by considering modularisation in biological networks [20] These methods attempt to first identify coherent groups of proteins and then assign functions to all of the proteins in each group. In terms of topology, a functional module can be typically understood as a group of proteins that are densely interconnected and contribute to perform for a specific biological function [21]. Once a module is obtained, the function prediction within the module [22] is usually conducted in a straightforward way by simple methods like orthology exploitation [10].

However, most of these approaches use PPI and, while very useful, they have limitations. The obtained PPI data result in a rich, but quite noisy and still incomplete, source of information. Also, PPIs are only available for a reduced group of organisms [15] due to the problems of using high throughput technologies in important study organisms such as *Mycoplasma*.

This last case implies a significant restriction since *Mycoplasma genitalium *is one of the most studied species, as it is the smallest organism having a small genome size [23,24] and has limited metabolic capabilities [25]. Due to all of the above, it has become a close approximation to the minimal set of genes required for bacterial growth [2,24], so, in order to study the Proteomes of these species, other type of protein-protein networks is necessary.

In many ways, Protein-Protein Association Networks (PPAN) are a more informative way of describing proteins and their mutual interactions [26]. In contrast to PPI, PPAN make no assertion as to how exactly two proteins interact: Proteins can show a productive functional interaction without physically interacting with each other (i.e. performing subsequent metabolic reactions in the same metabolic pathway). Therefore, whenever two proteins form a specific functional partnership, they can be thought of as being associated, independently of what the actual mechanism of their association is [26]. PPAN have been widely used recently in order to predict protein function [27-31].

In this work, a new protocol is described for predicting new gene annotations involving different approaches using PPAN for *Mycoplasmas*: Functional module identification, orthology exploitation and Gene Ontology (GO) Semantic similarity-based measures [32]. We have developed a simple, but effective, procedure in order to assign function transferring GO terms to unannotated protein nodes inside functionally conserved modules between two *Mycoplasma *species.

**Figure 1:**
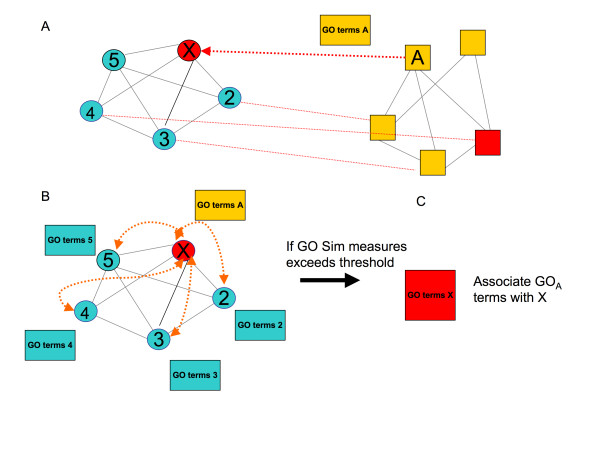
**Information theory-based semantic similarity (ITTS) for predicting function through interacting proteins**. A. If Orthology value exceed a threshold, we considered the protein pair between 2 functional modules as real orthologs and transfer GO terms specifically between annotated protein one species (yellow) to un-annotated × *M. genitalium *protein (red). B. Calculate semantic similarity between this GO terms assigned and GO terms from annotated neighbouring proteins inside the *M. genitalium *functional module. C. Consider GO terms A with similarity above a threshold, then associate protein × with GO. Orange dotted lines indicate Semantic similarity calculations.

The procedure is as follows: First, identify functional modules between two *Mycoplasma *PPAN (species A and B); second, assign Gene Ontology (GO) terms to each protein inside the species B modules *(*proteins without GO terms remain unannotated); third, calculate the orthology value between each potential pair of orthologs (A and B), and if the value exceeds a threshold, transfer GO terms specifically from Species B ortholog proteins to the Species A unannotated protein; Finally, the transferred GO terms list of unannotated Species A protein was analysed, comparing them with the annotated proteins GO terms inside the Species A module that they belong to, using GO semantic similarity measures. If the similarity value is above a threshold, then the Species A unannotated protein is associated with these assigned GO terms. A schematic view of the algorithm is depicted in Figure 1

## Results

### 1. Calculations and algorithm procedure

#### 1.2. Protein-protein association networks for *Mycoplasma *species

We have performed this study on seven *Mycoplasma *species proteomes. One of the main limitations in using protein-protein interactions for function prediction is the availability of interaction data, especially if one wishes to work with Mollicutes. The entries in STRING [33] correspond to protein-protein functional associations for more than 600 organisms, including *Mycoplasmas*. A protein-protein functional association can mean either a direct physical binding or an indirect interaction, such as participation in the same metabolic pathway or cellular process. The associations are derived from high-throughput experimental data, from the mining of databases and literature and from predictions based on genome context analysis. STRING carefully assesses and integrates all of these data in order to obtain a single confidence score for all protein interactions, taking a more generalised perspective on proteins and their associations than other databases whose main purpose is to collect and curate direct experimental evidence about protein-protein physical interactions.

Moreover, the improvement of the use of STRING in Protein Function network procedures has been indicated [15], and in a recent work, STRING has been used to study the proteome organisation of *Mycoplasma pneumoniae*.

**Figure 2:**
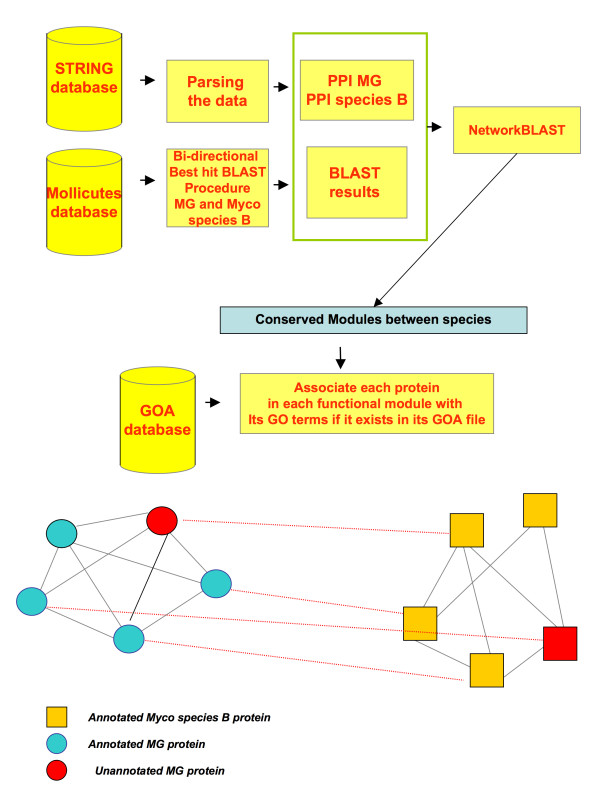
**Method procedure**. First, we obtained the PPANs for each of the two species from STRING database. Secondly, a BLAST bi-directional procedure is executed for both species. Then, a NetworkBLAST analysis using the PPANs and BLAST best hits results is executed and conserved functional modules between the two species are obtained. The GO terms for each protein node in the conserved modules are assigned using GOA files for each species. Proteins with no entry in GOA file remain unannotated.

The PPAN for each species were obtained from the STRING database (2008 release), the information for *Mycoplasmas *being extracted and then a Protein-Protein network for each of the species being constructed, resulting in undirected PPAN. The networks were built using high-associations (weighted score> 0.7). See Additional File 1 for all the *Mycoplasma *PPANs used in this study. These PPAN have been used for the following calculations. The following procedure is depicted on Figure 2.

#### 1.2. Functional Module identification

We first identified the functional modules shared between *M. genitalium *and every other Mycoplasma species using NetworkBLAST [22,34,35] as follows:

First, the proteomes in FASTA format were obtained for each *Mycoplasma *species from the GenBank repository dated October 2010 ftp://ftp.ncbi.nih.gov/genbank/, and for every two species analyzed, we performed a bi-directional BLAST [3] search of *M. genitalium *was performed over the other Mycoplasma's species 2 and vice versa. The algorithm was fed with the BLAST results and PPAN s of each of the species analyzed. NetworkBLAST outputs a set of modules that are evolutionarily conserved across the two PPAN. (See Table 1 for the number of interactions and functional modules detected between each genome and *M. genitalium*.)

**Table 1:** Number of interactions and functional modules detected for each genome and M. genitalium

Genome	Number of interactions	Modules detected
*M. penetrans*	37106	485

*M. pneumoniae*	22270	136

*M. hyopneumoniae*	18038	105

*M. capricolum*	28610	84

*M. mycoides*	19158	99

*M. pulmonis*	27365	81

#### 1.3. Gene Ontology (GO) terms assignation to proteins inside the modules

The standardized Gene Ontology vocabulary (GO) [36] was used as a standard to annotate the proteins inside the PPAN. The annotation for each gene in each proteome was obtained from Gene Ontology Annotation files (GOA) [37] available for each *Mycoplasma *proteome in the GO database http://www.geneontology.org. (See Table 2 for the total number of GO annotated genes in each genome). The filtered GO terms were associated for each node in each conserved module detected by NetworkBLAST. Each *M. genitalium *protein with no GO terms assigned in the GOA file is susceptible to be annotated by using its orthologs in the other species.

**Table 2:** Genomes, their number of genes and genes with GO annotations

Genomes	Number of genes	Number of GO annotations	GO annotations
*M. genitalium*	483	435	5124

*M. pneumoniae*	687	563	5809

*M. hyopneumoniae*	671	424	4670

*M. capricolum*	812	499	5697

*M. penetrans*	1028	629	5889

*M. mycoides*	978	711	6136

*M. pulmonis*	778	484	5355

#### 1.4. Transferring GO terms to unannotated protein

The orthology value calculated between each potential pair of orthologs between conserved modules using ORTHOMCL [38]. If the value exceed a threshold, the pair was considered as real orthologs and the procedure of Jaeger and Laeser [10] was followed for transferring GO terms specifically between those ortholog protein pairs and generating a list for the unannotated *M. genitalium *protein.

#### 1.5. Semantic similarity analysis of transferred GO terms

The transferred GO annotations list of unannotated *M. genitalium *proteins was analyzed, comparing them with the GO annotations from annotated proteins inside the module that they belong to. The Information theory-based semantic similarity (ITTS) for predicting function through interacting proteins was followed [10,14]:

• To calculate Semantic Similarity between the transferred GO terms from unannotated protein and GO terms from interacting neighbours inside the same functional module.

• To consider GO terms with a similarity value above a threshold, then associate unannotated protein with these GO terms

However, we can accept the predictions if they are similar to the annotations in the module, i.e., have similar GO ontology annotations, then a "manual curation" procedure is needed. A schematic view of the ITTS procedure is depicted in Figure 1.

### 2. Performance measures

#### 2.1. Effectiveness of the method

The performance of the method was measured as an average value in a five-fold cross-validation analysis, where the GOA dataset for *M. genitalium *was randomly divided into five parts. Four parts for model learning (training), and the remaining part for validation (testing). Known GO annotations were removed from the test set and it was tried to predict the terms of the proteins in the test set using the rest of the sets (training sets).

Finally, the predicted terms were compared with original annotations to determine the amount of correctly predicted annotations. Effectiveness is validated using standard information retrieval measures: recall and precision. Several terms have defined:

A: set of annotated GO functions (in test set)

P: set of predicted GO functions

F: GO functions in train set

So, we can establish:(1)(2)(3)(4)

then, define:(5)(6)

The variance of the reconstructed annotation was computed in order to see if it is affected by the random split choice. The performance of the method was measured as an average value in a five-fold cross-validation analysis using each of the *Mycoplasma *genomes (*M. penetrans*, *M. pneumoniae*, *M. capricolum*, *M. hyopneumoniae*, *M. pulmonis *and *M. mycoides*) to predict the *M. genitalium *annotations for two ontologies: Biological Process and Molecular Function. These genomes vary greatly in the availability of annotations and interaction data, which provides a good setup to study the strengths and limitations of our procedure. As shown in Table 3 for Molecular Function ontology, the average of recall is nearly 90% in all species (except *M. pneumoniae *which reaches 98% and, on the other hand, *M. pulmonis *only reaches 81.5%) and the average of precision is 65%. On the other hand, the average of precision is 30% and the average of recall is 80% in the Biological Process ontology. As expected, recall is higher in the Molecular Function ontology due to, in previous studies, researchers having found that sequence similarity is strongly correlated with semantic similarity based on the Molecular Function aspect of GO [39]. This aspect fits with the biological expectations. The sequence of a protein determines its molecular function but does not necessarily relate to the biological process that it is involved in.

**Table 3:** The performance of the method measured as an average value in a 5-fold cross-validation analysis for two ontologies: Biological Process and Molecular Function.

PROTEOME	AVERAGE PRECISION		AVERAGE RECALL	
	
	BP	MF	BP	MF
*M. hyopneumoniae*	0,305	0,661	0,815	0,879

*M. pneumoniae*	0,331	0,653	0,987	0,987

*M. penetrans*	0,317	0,635	0,899	0,895

*M. mycoides*	0,309	0,655	0,793	0,837

*M. capricolum*	0,322	0,643	0,928	0,921

*M. pulmonis*	0,306	0,664	0,754	0,815

Average Precision and Average Recall values for the 5 folds in each genome and for each ontology is presented. Left side Biological Process, right side Molecular Function.

The high recall in both ontology cases indicates that a large number of GO terms is recovered by our method from all of the GO terms that are relevant to the search. The high precision in the Molecular Function ontology case indicates that a large proportion of the GO terms are relevant to the search among all of the GO terms recovered by our method.

#### 2.2. Predictions in the *M. genitalium *dataset using different GOA files

Two GOA files for *M. genitalium *were used in this study. The GOA2005 in this article is dated March 2005, and was obtained directly from NCBI. GOA2005 contains 443 distinct gene-GO entries. The second GO annotation file, referred to as GOA2010, is dated October 2010 and was obtained from NCBI also. Table 4 summarises the content of both files. It is seen that the two files are consistently similar in terms of GO annotations for each gene (i.e., a similar number of genes with more than 10 annotations...) despite the number of annotation gaps between them.

**Table 4:** Content of GO terms and genes for each Gene Ontology Annotation (GOA) file used in our experiments.

Database			GO terms	
	
	Total	>10 annotations (%)	3-9 annotations (%)	<2 annotations (%)
GOA2005	443	36(9,7%)	56 (12,6%)	263(59,36%)

GOA2010	483	46(9,3%)	60 (12,15%)	291(58,9%)

See text for details

**Figure 3:**
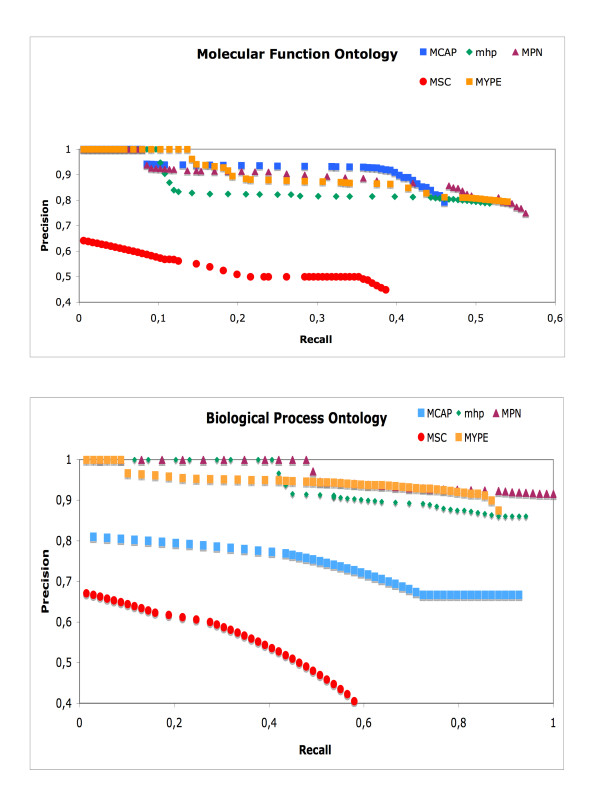
**Precision and Recall values for Resnik semantic similarity measure using GOA2005 file**. The performance of the method using each of the *Mycoplasma *genomes (*M. penetrans, M. pneumoniae*, *M. capricolum*, *M. hyopneumoniae*, *M. pulmonis *and *M*. *mycoides*) to predict the *M. genitalium *annotations for two ontologies: Biological Process and Molecular Function, see Methods for details. The cut-off values of semantic similarities were varied to generate the different data points on the curves.

In order to determine the accuracy of the predictions in real-world conditions where most genes are poorly annotated or not at all (less than 10 gene annotations per GO term), the same procedure as above was followed (using each of the *Mycoplasma *genomes to predict the *M. genitalium *annotations), but now using the older GO2005 file and then validating the newly predicted annotations using the newer GOA2010 association file as a second evaluation. Using a similar procedure as Tao's [14], those GO terms marked as 'obsolete' and the ambiguous terms 'Biological Process unknown', 'Molecular Function unknown' were excluded from GOA2005. We limited our testing dataset to include only those GO terms that had at least three associated genes [14]. Two separate experiments were conducted: one to predict annotations for the Molecular Function ontology and another for the Biological Process ontology. The semantic similarity was calculated using different measures according to Couto [32] (See Methods for details). The cut-off values of semantic similarities were varied to generate the different data points on the curves. The results are shown in Figure 3. Due to space constrictions, only *M. genitalium *predictions using *M. pneumoniae *results are presented here. However, the rest of the results can be obtained from the authors upon request (See Additional File 2).

We found that our procedure performed well, for the Molecular Function Ontology, and high precision scores (more than 80%) and recall values of nearly 60% were obtained. In the Biological Process Ontology, precision scores obtained were also high (more than 90%) and recall values of nearly 85% were obtained for those GO terms that were associated with two or more genes. However, it can be seen that in both experiments (Molecular Function and Biological Process Ontology) poor values were obtained compared with the other genome predictions in the case of *M. mycoides*. We believe that the cause of these results could be that the higher semantic similarity values obtained for all of the GO terms predicted and those that are part of the modules are barely 0.6 (data not shown), while the higher semantic similarity values for the other genomes oscillate between 0.7 and 0.85.

#### 2.3. Predictions of informative GO terms in the *M. genitalium *dataset using different approaches

We also, wish to determine if our method can be compared with other similar method in terms of precision and recall when trying to predict informative GO terms. Informative GO terms were defined as those terms that are annotated to at least n proteins (n = 10) and has at least a level-4 or higher. Defining:

t: Relevant/Informative GO terms

n: Retrieved GO terms(7)

and define now Precision and Recall as:(8)(9)

Here, how well our procedure detects informative GO terms, as compared to other similar approximations is studied. FS-Weight is an algorithm which predicts the function of a protein based on the idea that the interaction between indirect neighbours inside a PPI is likely to share common functions [9]. These indirect neighbours may interact with the same protein due to some common physical or biochemical characteristics, especially if they share many common interaction neighbours. The FS-weight algorithm was chosen, from others for several reasons: First, because the method is one of the latest PPI-based algorithms for predicting protein function using GO annotations. Secondly, because it was tested to predict protein function using the STRING database, showing significant improvement and, thirdly, because FS-Weight can predict protein function effectively for all of the three categories of GO across different genomes, indicating that it is a robust approach [15]. We also compared our procedure with two other approximations: The first one [6] attempts to learn a linear model of how likely a protein is to have a function given the frequency with which a proteins neighbours have that term. Parameters of the model are estimated using quasi-likelihood estimation techniques. In order to provide a fair comparison, the proposed method has been compared to a sequence based annotation method which is based on the transfer of annotations in a closely related species, which is BYPASS [5]. This method predicts the putative function for the protein from its sequence integrating the results from the PSI-BLAST programme and a fuzzy logic algorithm using several protein sequence characteristics which have been checked, with regards to their ability to rearrange a PSI-BLAST profile according more to their biological functions.

**Figure 4:**
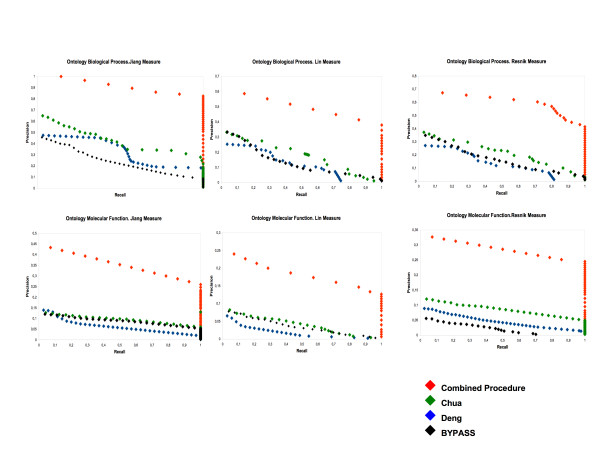
**Predictions of informative GO terms in the *M. genitalium *dataset using different approaches**. The Precision versus Recall graphs for the prediction of informative GO terms Biological Process (upper) and Molecular function (lower side) Ontology using different GO semantic similarity measures for our combined procedure and FS-Weight.

We wish to study the prediction performance of our procedure for detecting conserved modules among *Mycoplasma *species. Due to space constrictions, only *M. genitalium *and *M. pneumoniae *results are presented here, however, the rest of the results can be obtained from the authors upon request. The precision versus recall graphs for the prediction of informative GO terms are depicted in Figure 4 for both Molecular function and Biological Process Ontology.

Different semantic similarity measures were used to obtain scores between the GO terms included inside of each predicted module and the GO terms obtained using our procedure. For PPI predictions, the modules obtained were assigned using NetworkBLAST and then calculated the semantic similarity between the GO terms included and the GO terms predicted. For BYPASS predictions, the GO terms were assigned to the predictions using the Gene Ontology database directly. The semantic similarity values were then used as variable thresholds. (See Material and Methods for details).

It can be seen in Figure 4 that, for the three semantic similarity calculations our algorithm makes predictions with better precision and recall, as compared to PPI (Chua and Deng) and sequence-analysis methods. The main advantage of our procedure is that, despite obtaining a low number of predicted GO terms, they are related to the terms inside of each module, while the PPI predictions use only one PPAN (in this case the *M. genitalium*) and, then, the predicted GO terms have lower similarity values than do our predictions. It can be seen, however, that our results have the same problem as do the PPI aproximations [15]. Due to the lack of annotation information and our previous parsing in trying to avoid inconsistency in GO terms, the informative terms chosen are a low number (nearly 50) and may not provide statistically strong comparisons.

Finally, the number of *M. genitalium *unnanotated genes with function predictions, with respect to GO annotations, can be found in Table 5. Using our method, not only general functional categories are assigned to unannotated proteins, but they are also assigned very specific functions to unannotated proteins (complete results are available upon request to the authors). Predictions are consistent with effectiveness results. These genomes vary greatly in the availability of annotations and interaction data. As shown in Table 5 the coverage of annotations vary from 17% to nearly 70%, for the species that have more functionally conserved modules with *M. genitalium*, *M. pneumoniae*, *M. hyopneumoniae *and *M. penetrans*.

**Table 5:** *M. genitalium *using different genomes and number of genes unannotated. Coverage of annotations using our method (Predictions over number of unnanotated genes)

Genomes	Coverage of annotations	Number of GO terms assigned
*M. pulmonis*	0.23	120

*M. pneumoniae*	0.69	535

*M. hyopneumoniae*	0.66	470

*M. capricolum*	0.17	163

*M. penetrans*	0.57	460

*M. mycoides*	0.27	155

## Discussion

It has been shown herein, that our procedure out-performs other similar algorithms to predict GO-based annotations using Protein-Protein networks, with equal or higher overall precision from a significantly broader range of GO terms. The incorporation of other approximations such as functional module detection, conserved between species and orthology exploitation, predict function with higher precision and recall in two ontologies of the GO database. As compared to other GO search engines, our algorithm is capable of finding GO terms with high semantic similarity values due to using orthology information between proteins predicted inside functional modules conserved between species, and it has been also shown to recapitulate "known" future GO annotations artificially removed from the dataset using five-fold cross-over validation, with high precision and recall.

## Conclusion

We believe that our combined approach could be applied in future as a high-precision Mollicute genome annotation procedure: Moderately well GO-annotated *Mycoplasma *genomes could help to improve protein function in other *Mycoplasma *less-annotated genomes in a more effective way than *ab initio *classic annotation methods. However, caution must be exercised when using this technique. We have shown how critical neighbour genomes with good GO annotation are: The performance of this procedure is limited because it needs *Mycoplasma *genomes with a high GO annotation degree and a high number of predicted conserved modules. If genomes were used with low predicted modules and a low number of GO terms annotated within those modules, a reliable number of predictions could not be achieved (data not shown). Future work will cover this by using the latest development of the GO database, and which evolutionary distances between Mollicute genomes are still allowable in order to predict a reliable number of conserved functional modules between them.

## Methods

### Gene Ontology annotation file

The standardised Gene Ontology vocabulary (GO) was used [36] as a standard to annotate the proteins inside the PPAN. The annotation for each gene in each proteome was obtained from the Gene Ontology Annotation file (GOA) [37] available for each *Mycoplasma *proteome in the GO database http://www.geneontology.org. The version of GO used in this study dated from October, 2008, and was parsed for each of the species, thus avoiding several problems:

• Excluding terms annotated as obsolete and terms that no have relation with *Mycoplasma *such as: GO:0000004. (Biological process unknown).

• Nearly all *Mycoplasma *proteomes are annotated in GOA files mainly with GO terms with evidence code IEA (Inferred from Electronic Annotation). To be retained, IEA annotations must be manually reviewed in order to be assigned an upgraded Evidence Code such as ISS (Inferred from Sequence or Structural Similarity). All the of IEA GO terms that do not have an associated ISS code were removed.

• IEA annotations are generalised to apply to a diverse range of species and usually only represent very broad functions such as 'Protein binding' and 'Enzyme binding'. In effect, this means that as functional genomics data are modelled using GO annotation, a large proportion of the remaining data describes only very broad biological concepts [40]. GO terms are arranged in a hierarchical manner with more general terms at the lower level and more specific terms at the higher level. The GO term "biological process" is defined as level 0, its children terms as level 1, and so on. Wong's work was followed and only GO terms were considered to contribute to "annotated protein" if they had at least one level-4 GO term or higher.

### GO semantic similarity calculations

The semantic similarity between two concepts has been used for investigating the relationships between GO annotations and gene sequences [13,39] as well as in clustering genes functionally [19].

The information content of each GO term for each protein inside the detected modules was calculated and then three measures were applied to estimate the semantic similarity between GO terms assigned using orthology exploitation to un-annotated proteins and the GO terms assigned to the annotated proteins inside the same functional module. Information content is defined as the frequency of each term which occurs in the GO corpus.

The semantic similarity of one GO term *go1 *and a GO terms set *GO *= {*go*_1_,*go*_2_...*go_k_*} is dfined as:(10)

As GO allows for multiple parents for each concept, two GO terms can share parents by multiple paths. We take the minimum *p(go)*, where there is more than one shared parent. *p_ms _*is defined as;(11)

where *S*(*go*_1_, *go*_2 _) is the set of parent terms shared by *go*_1 _and *go*_2_.

Different similarity calculations were then followed depending upon the GO ontology. For the Molecular Function ontology, Lord's procedure [39] was followed and the Resnik Similarity measure was applied [41]:(12)

For the Biological Process ontology, we followed Couto [32] and Lin was applied [42](13)

and also, Jiang [43] measure(14)(15)

Therefore, given two GO terms sets, one for the annotated protein *GO*_*ann *_= {*go*_*ann*1_, *go*_*ann*2_...*go*_*annk*_} and another for the Functional module *GO *= _mod_{*go*_mod1_, *go*_mod 2 _...*go*_mod _*_k_*}, the semantic similarity between them is defined as:(16)

Depending on the similarity between the GO annotations for the protein and the GO annotations for the module which it belongs to, this score ranges from between 0 and 1, where 1 indicates functional equality and 0 indicates maximal functional distance. Low values can be discriminated among, indicating low precision, and higher values, indicating high precision.

## Abbreviations

PPI: Protein-protein interaction; PPAN: Protein-Protein Association Networks; GO: Gene Ontology; IEA: Inferred from Electronic Annotation.

## Competing interests

The authors declare that they have no competing interests.

## Authors' contributions

EQ and AG conceived the study. AP participated in conceptualization and discussion. AG and JC designed the main algorithm and developed the PERL STRING processing scripts and R Similarity calculations scripts. IA performed the BLAST and functional modules detection. JP made the performance measurements. AG drafted the manuscript. All authors read and approved the final manuscript.

## Supplementary Material

Additional file 1 **PPANs for each Mycoplasma genome extracted from STRING**. Each sheet corresponds to one PPAN *Mycoplasma *genome (M. Genitalium, *Mycoplasma genitalium*, M. Gallisepticum: *Mycoplasma gallisepticum*, MPN: *Mycoplasma pneumoniae*, MYPE: *Mycoplasma penetrans*, MYPU: *Mycoplasma pulmonis*, Synoviae: *Mycoplasma synoviae S53*, MMOB: *Mycoplasma mobile *MCAP: *Mycoplasma capricolum*, mhp: *Mycoplasma hyopneumoniae*) The sheets are tabulated in two columns (the two interacting proteins that have a functional association as described in STRING). The STRING scores are not described in this file but can be available from authors upon request. Click here for file

Additional file 2 ***M. genitalium *predictions using *M. pneumoniae *PPAN**. The file is tabulated in four columns: *M. genitalium *ORF, Number of functional module conserved between *M. genitalium *and *M. pneumoniae*, Resnik GO Similarity Measure between GO terms list assigned from *M. pneumoniae *ortholog and GO terms from neighbor *M. genitalium *protein inside its functional module, List of GO terms assigned to the protein. Click here for file
